# Uterine torsion with degeneration and infarction of giant leiomyoma in a postmenopausal woman: A case report

**DOI:** 10.1097/MD.0000000000035964

**Published:** 2023-11-10

**Authors:** Zhongxue Ye, Yafen Jiang, Kun Yan, Chen Yu

**Affiliations:** a Department of Gynecology, Ningbo No. 2 Hospital, Ningbo, China; b Department of Radiology, Ningbo No. 2 Hospital, Ningbo, China.

**Keywords:** giant leiomyoma, postmenopausal, uterine torsion

## Abstract

**Rationale:**

Uterine torsion and giant leiomyoma are both rare diseases. Uterine torsion combined with giant leiomyoma with degeneration or infarction is easy to be misdiagnosed. We wrote this case to increase the accuracy and timeliness of medical staff’s diagnosis and treatment of uterine fibroids combined with uterine torsion.

**Patient concerns:**

We present a case of uterine torsion with degeneration and infarction of giant leiomyoma in a 66-year-old postmenopausal woman, who had a lump in her pelvis 10 years ago and suffered from acute abdominal pain half a day before hospitalization.

**Diagnosis:**

The patient was considered as uterine torsion with huge abdominal mass by computed tomography and enhanced magnetic resonance imaging, and finally diagnosed as uterine torsion with giant leiomyoma through surgery and pathological examination.

**Interventions and outcomes:**

The patient underwent exploratory laparotomy. In addition to the removal of huge uterine fibroids, the hysterectomy with double appendages was conducted. The histopathologic analysis showed “(Uterine tumor) leiomyoma with extensive edema, degeneration, infarction and calcification.” The patient recovered well after operation and kept healthy in the follow-up to date.

**Lessons:**

Although uterine torsion is extremely rare, early diagnosis and treatment are essential to prevent serious complications.

## 1. Introduction

Uterine fibroids are the most common benign tumors in gynecology, affecting more than 70% of women worldwide.^[1]^ Common clinical symptoms include abnormal menstruation, abdominal mass, abdominal pain, and compression symptoms such as changes in urine and bowel movements. The definition of giant uterine fibroids is not completely clear. Clinicians consider that the diameter >10 cm or weight >11.4 kg^[2]^ can be called giant uterine fibroids, which is relatively rare in clinical practice. Due to the huge size of the tumor, it is easy to occur ischemia, necrosis, and cystic degeneration, which increases the difficulty of differentiation from uterine sarcoma and ovarian tumor.

Uterine torsion is an extremely rare condition, which means a rotation >45 degrees around the long axis of the uterine body.^[3]^ Common causes include pregnancy, giant fibroids, and ovarian cysts. It is reported in female at any age and is more common in women of reproductive age. The clinical manifestations of uterine torsion include abdominal pain, nausea, vomiting, and urinary symptoms. Uterine torsion is often missed or misdiagnosed because of atypical clinical symptoms. Computed tomography (CT) or magnetic resonance imaging (MRI) examination is helpful for the diagnosis of uterine torsion. The typical imaging findings include helical changes of uterus (or “X” shaped) and torsional jumping of uterine vessels.^[4]^ Here, we report a rare case of uterine torsion with degeneration and infarction of giant leiomyoma from Ningbo No. 2 Hospital in July 2022.

## 2. Case report

**Figure 1.**
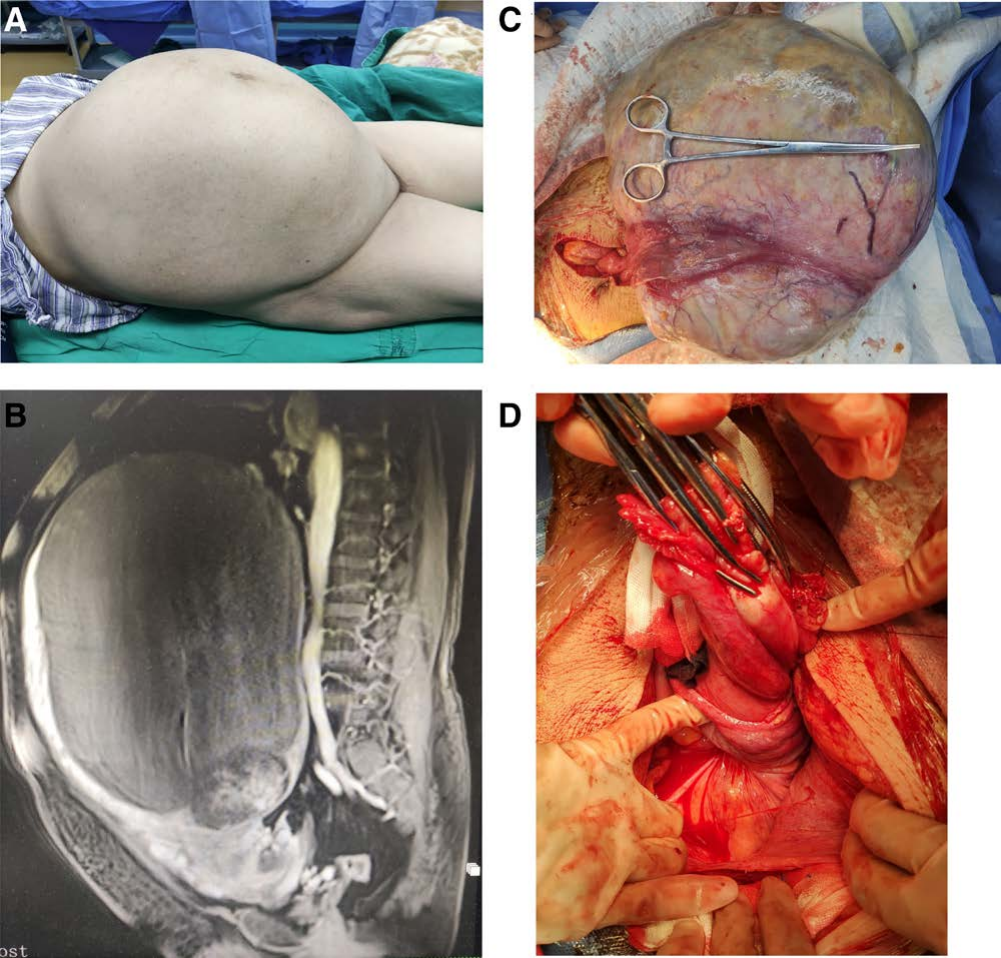
Photos taken from the patient showing the giant mass and torsion uterus. (A) Distended abdomen of the patient. (B) Enhanced MRI showed the giant mass and torsion uterus. (C) Giant mass out of the abdominal cavity. (D) Torsion uterus. MRI = magnetic resonance imaging.

A 66-year-old postmenopausal woman was admitted for treatment of abdominal mass and pain. The patient gave written informed consent to publish this case report. She had a history of 2 pregnancies and 1 birth. Ten years ago, the patient found a pelvic mass about 3 cm in size by B-ultrasound examination, without discomfort, and no treatment and follow-up were made. After that, the patient felt that the abdomen was gradually swollen. One year ago, the patient began to have intermittent dull pain in the lower abdomen, which was mild, aggravated when she was tired, and could be relieved by lying flat. Half a day before admission, there was persistent pain in the lower abdomen, which was severe and accompanied by sweating. Physical examination found that the abdomen was protuberant. There was a huge mass in the abdomen, which went up to the xiphoid process and down to the pelvis. The left and right sides reached the midaxillary line. The movement was poor, and the tension was high, with tenderness in the lower part of the mass. The palpation of the uterus and bilateral accessories was not satisfactory (Fig. 1A). The abdominal CT scan showed that “(1) A huge mixed solid/cystic mass in abdomen about 30 cm × 28 cm × 11 cm in size. (2) The cervix was deformed and twisted, uterus torsion? (3) Subcutaneous edema of pelvic and abdominal wall, and a small amount of pelvic effusion.” Contrast-enhanced MRI suggested that “(1) A huge mixed solid/cystic mass in abdomen about 30 cm × 28 cm × 11 cm in size, considering leiomyoma with degeneration, or benign or low-grade malignant tumor from accessory origin, cystadenoma? (2) Uterus torsion” (Fig. 1B). Laboratory tests showed a CA125 level of 73.3 U/mL. Following preoperative evaluation, exploratory laparotomy was scheduled.

During the operation, about 50 mL faint yellow effusion was observed in the abdominal cavity. A huge mixed solid/cystic mass about 30 cm × 25 cm × 16 cm was found in the abdominal cavity, and the surface of the mass was smooth, which seemed to be connected with the uterus. After aspirating 5000 mL of coffee colored fluid through puncture, the tumor was delivered out of the abdominal cavity (Fig. 1C). It was found that the tumor came from the left anterior wall of the uterus, and the uterus twisted 540° (Fig. 1D). No obvious necrosis was discovered of the uterus. The tumor was removed and the intraoperative pathological detection indicated “(Uterine tumor) benign cystic disease.” No other lesions were found in the pelvis and abdomen and the hysterectomy with double appendages was conducted. The final histopathologic analysis showed “(Uterine tumor) leiomyoma with extensive edema, degeneration, infarction and calcification.” The patient recovered well after operation, and no abnormality has been found in the follow-up up to now.

## 3. Discussion

Uterine torsion is a very rare disease, which generally noted in gravid uteri because of the relaxation of pelvic structure and laxity in pregnancy.^[5]^ However, enlarged fibroids are the most common predisposing factor related to uterine torsion in nonpregnant women as the weight of the fibroid makes an asymmetric weight distribution.^[6,7]^ Less than 10 cases of uterine torsion in postmenopausal women were reported in the past 10 years, among which, 70% of the patients were accompanied by giant leiomyoma.^[8]^ Normally, the uterus is firmly stabilized and attached to the pelvic cavity through the ligaments. Under the traction of huge leiomyoma, as well as the loss of collagen and elastin with the growth of age, the uterine ligaments are progressively stretched, with the firmness reduced. This may affect the stability of the uterus, resulting in rotation of the uterus.

The most common clinical manifestation of patients with uterine torsion is acute intense abdominal pain, which is consistent with our patients. The difference is that our patient had chronic abdominal pain for 1 year, which may be attributed to her giant leiomyoma. Some patients may also have ascites. However, there are also some patients whose symptoms are not obvious, or the abdominal pain is gradually relieved, just like our patient, whose abdominal pain was significantly relieved on the second day of admission. This may be related to uterine torsion without affecting its blood supply.

In addition to clinical manifestations, imaging examination plays an important role in the diagnosis of uterine torsion, including CT and MRI. Compared with CT, we suggest MRI, especially enhanced MRI, has more advantages in the diagnosis of uterine torsion, as MRI has better resolution in soft tissues. Characteristic findings include spiral changes of the uterus, or “X” shape, and the uterine blood vessels can be seen jumping from left to right by enhanced examination.^[9]^ In this case, imaging experts noted the spiral changes of the uterus and changes in vascular routing, supporting the diagnosis of uterine torsion. In addition, MRI is also helpful in the diagnosis of giant leiomyoma. However, CT is also a pretty good choice for patients with metal in the inner body. With patients who have a medical history of kidney failure or hypersensitivity to contrast media, dynamic review of plain CT is also an effective diagnostic method.^[8]^

What limits us is that we cannot ascertain the source of the huge abdominal mass before surgery. In addition to the symptoms, physical examination, and imaging examination, previous medical history is also very important. The patient reported having a 3 cm pelvic mass 10 years ago, but unfortunately, the medical records were not clear and the examination form was lost. The loss of previous medical information is very common for patients. The online upload of medical information in Ningbo, China will be beneficial for the overall health management of patients.

The treatment of nonpregnant uterine torsion with huge pelvic or abdominal mass is mainly surgery. The specific operation method depends on the patient’s age, basic conditions, fertility requirements, and whether the uterus has ischemic necrosis. If serious complications such as hemorrhage, sepsis, and coagulopathy have occurred, active medical treatments are required. Considering the fetal factors, the diagnosis and management of uterine torsion during pregnancy are more difficult, which will not be detailed in this article.

## 4. Conclusion

Here we reported a postmenopausal woman diagnosed as uterine torsion with degeneration and infarction of giant leiomyoma. The patient presented with a huge abdominal mass accompanied by acute abdominal pain. Through inquiring about her medical history, physical examination, and CT and MRI examinations, we considered that she had uterine torsion, and the huge mass was considered as a source of the reproductive system. Through exploratory laparotomy, we determined that the patient had a huge uterine fibroid with uterine torsion. We removed her uterus and double appendages, and the patient recovered well after the surgery.

To sum up, uterine torsion is a rarely seen disorder. Large uterine fibroid is an important cause of uterine torsion, and CT or MRI is a significant examination for the diagnosis. Surgery is the main treatment for uterine torsion combined with pelvic or abdominal mass. Although uterine torsion is very rare, early diagnosis and treatment are essential to prevent serious complications.

## Author contributions

**Conceptualization:** Zhongxue Ye.

**Data curation:** Zhongxue Ye, Kun Yan, Chen Yu.

**Formal analysis:** Zhongxue Ye.

**Supervision:** Yafen Jiang.

**Writing – original draft:** Zhongxue Ye.

**Writing – review & editing:** Yafen Jiang, Kun Yan.
